# A Novel Validated Recurrence Stratification System Based on ^18^F-FDG PET/CT Radiomics to Guide Surveillance After Resection of Pancreatic Cancer

**DOI:** 10.3389/fonc.2021.650266

**Published:** 2021-05-12

**Authors:** Miaoyan Wei, Bingxin Gu, Shaoli Song, Bo Zhang, Wei Wang, Jin Xu, Xianjun Yu, Si Shi

**Affiliations:** ^1^ Department of Pancreatic Surgery, Fudan University Shanghai Cancer Center, Shanghai, China; ^2^ Pancreatic Cancer Multidisciplinary Center, Fudan University Shanghai Cancer Center, Shanghai, China; ^3^ Department of Oncology, Fudan University Shanghai Medical College, Shanghai, China; ^4^ Pancreatic Cancer Institute, Fudan University, Shanghai, China; ^5^ Shanghai Pancreatic Cancer Institute, Shanghai, China; ^6^ Department of Nuclear Medicine, Fudan University Shanghai Cancer Center, Shanghai, China; ^7^ Center for Biomedical Imaging, Fudan University, Shanghai, China

**Keywords:** ^18^F-FDG PET/CT, radiomic features, pancreatic cancer, recurrence, surveillance

## Abstract

**objective:**

Despite the heterogeneous biology of pancreatic cancer, similar surveillance schemas have been used. Identifying the high recurrence risk population and conducting prompt intervention may improve prognosis and prolong overall survival.

**Methods:**

One hundred fifty-six resectable pancreatic cancer patients who had undergone ^18^F-FDG PET/CT from January 2013 to December 2018 were retrospectively reviewed. The patients were categorized into a training cohort (n = 109) and a validation cohort (n = 47). LIFEx software was used to extract radiomic features from PET/CT. The risk stratification system was based on predictive factors for recurrence, and the index of prediction accuracy was used to reflect both the discrimination and calibration.

**Results:**

Overall, seven risk factors comprising the rad-score and clinical variables that were significantly correlated with relapse were incorporated into the final risk stratification system. The 1-year recurrence-free survival differed significantly among the low-, intermediate-, and high-risk groups (85.5, 24.0, and 9.1%, respectively; p < 0.0001). The C-index of the risk stratification system in the development cohort was 0.890 (95% CI, 0.835–0.945).

**Conclusion:**

The ^18^F-FDG PET/CT-based radiomic features and clinicopathological factors demonstrated good performance in predicting recurrence after pancreatectomy in pancreatic cancer patients, providing a strong recommendation for an adequate adjuvant therapy course in all patients. The high-risk recurrence population should proceed with closer follow-up in a clinical setting.

## Introduction

Pancreatic ductal adenocarcinoma (PDAC) is a fatal malignancy with a 5-year overall survival less than 9% (1). Early detection of pancreatic cancer remains challenging; radical surgical resection offers the only chance of cure, and at best, only 20% are suitable for curative resection in newly diagnosed patients (2). Despite considerable improvements in surgery, the overall survival (OS) of these resected cases remains poor, and the recurrence rate is 80%. The previous study identified a recurrence-free interval of 12 months as the optimal cutoff in pancreatic cancer to distinguish early and late recurrence (3). Data on the roles of surveillance in patients with resected PDAC are limited, and similar surveillance schedules are applied after resection for all pancreatic cancers despite their heterogeneous biology. Specifically, carbohydrate antigen 19-9 (CA19-9) measurements and contrast-enhanced computed tomography (CT) every 3 to 6 months for 2 years after radical resection are recommended according to the latest version of the National Comprehensive Cancer Network (NCCN) guidelines. The expert opinion of French clinical practice guidelines proposed that the evaluation and surveillance of patients after curative resection to monitor relapse at an early stage should be performed every 3 months over 2–3 years, and then every 6–12 months up to 5 years (4). Therefore, establishing a recurrence stratification system to guide surveillance of resected pancreatic cancer (RPC) patients is urgent need in this field.

In diagnosing of pancreatic cancer, CT is the first-line imaging approach used to determine the resectability according to the NCCN criteria to predict R0 resection. Secondary signs, including pancreatic duct dilatation, are vital to diagnose PDAC (5). Pancreatic cancer cells have extensively reprogrammed metabolism, and the most useful aspect of ^18^F-positron emission tomography/CT (^18^F-PET/CT) is that it adds precise anatomical localizations to functional data. Thus, ^18^F-PET/CT plays superior diagnostic roles in evaluating the stage, determining the therapy response, predicting survival and detecting recurrence compared with conventional imaging (6–9). Its value in predicting distant metastasis and survival likely originates from the strong correlation between the levels of fluorodeoxyglucose (FDG) uptake and tumor aggressiveness in terms of the pathological grade (10). For resected PDAC patients, ^18^F-FDG PET/CT metabolic parameters, including the maximum standardized uptake value (SUVmax), total lesion glycolysis, and metabolic tumor volume, are significantly associated with OS or disease-free survival (11), indicating that PET/CT scan activity may act as a prognostic factor after pancreatectomy, consistent with the conclusion that glucose metabolic pathways are crucial in PDAC biology.

As a non-invasive, data-characterization algorithms that assesses the spatial heterogeneity of volumes of Interest (VOIs) in medical imaging, texture analysis of tumors has attracted increased interest (12). Radiomics has emerged in this context and is the most advanced in application within the medical field of oncology, which extracts high-throughput features from radiographic medical images and provides insight into the underlying innate biology of tumors. These features, termed radiomic features, may be useful for improving the predictive accuracy and therapeutic response for various conditions of the disease, potentially uncovering the valuable disease characteristics for personalized therapy (13). The radiomic features of PDAC have been investigated recently, identifying prognostic intratumor heterogeneity or predicting survival intervals and treatment responses based on CT, MRI, or PET/CT radiomic features (14–18), which could optimize treatment strategies and facilitate individualized therapy in this field. Here, we postulated that combining ^18^F-FDG PET/CT radiomic features and clinicopathological characteristics could reflect the properties of pancreatic tumors and may provide valuable information to improve prognostic prediction. Therefore, this study aimed to establish a recurrence risk stratification system for initially resectable PDAC patients after pancreatectomy to better guide monitoring and surveillance in a clinical setting.

## Materials and Methods

### Patient Selection

Consecutive resectable patients with histopathologically confirmed PDAC who had undergone preoperative ^18^F-FDG PET/CT followed by radical pancreatectomy between January 2013 and December 2018 at the Department of Pancreatic Surgery, Shanghai Cancer Center, were included. The criteria defining the resectability status at diagnosis were made by multidisciplinary discussions based on the NCCN guidelines. Additionally, all the patients in the cohort had undergone a pancreatic protocol CT for staging and the determination of local resectability. Only initially resectable patients were included in this study.

^18^F-FDG PET/CT imaging was performed within 15 days before surgery and without antitumor treatment received within at least 2 years before the examination. The exclusion criteria of our study were as follows: (1) malignancy other than pancreatic cancer was present; (2) a tumor site with low-grade ^18^F-FDG uptake (less than 2.5); (3) the primary tumor was too small for accurate texture analysis; (4) an R2 surgical margin; and (5) postsurgical radiotherapy. The data collected included patient demographics, PET/CT slices, metabolic activity parameters, CA19-9 levels, pathological parameters, adjuvant therapy regimen and number of cycles. Specifically, preoperative CA19-9 was measured within 7 days before surgery and 4–6 weeks postoperatively. Recurrence in our cohort included both local and distant disease relapses.

One hundred fifty-six PDAC patients were enrolled according to the above criteria, and the patients were divided into two cohorts based on the time of consultation, with 109 patients assigned to the development set (from 2013.01 to 2016.12) and 47 assigned to the validation set (from 2017.01 to 2018.12). The study was approved by the institutional ethics committee.

### ^18^F-FDG PET/CT Scan Protocol and Image Acquisition

^18^F-FDG PET/CT scans were performed using a Siemens CTI RDS Eclipse ST system (Knoxville, Tennessee, USA). The enrolled patients were asked to fast for at least 6 h and presented blood glucose levels less than 8 mmol/L at the injection of ^18^F-FDG (7.4 MBq/kg body weight) intravenously. Image acquisition started 60 ± 5 min following the tracer administration. The CT acquisition parameters were as follows: tube voltages: 120 kV, tube current: 80–250 mA, slice thickness: 5.0 mm, pitch: 1.0 mm, rotation time: 0.5 s. PET was acquired with 2–3 min per table position. PET image data sets were reconstructed iteratively using an ordered-subset expectation maximization iterative reconstruction (OSEM) by applying CT data for attenuation correction. The reconstruction parameters were as follows: iterations: four, subsets: eight, pixel size: 4.0 × 4.0 mm, zoom: 1.0, full width half maximum (FWHM): 6.0 mm, and slice thickness: 5.0 mm. PET scanning and a low-dose CT scans were then performed immediately. The PET images were acquired using CT-based attenuation correction. The ordered-subsets expectation maximization technique was adopted to reconstruct the PET data—specifically, a 168 × 168 image matrix with eight subsets and four iterations. Coregistered scans were displayed on a workstation.

### VOI Drawing, Radiomic Feature Extraction and Image Analysis

**Figure 1:**
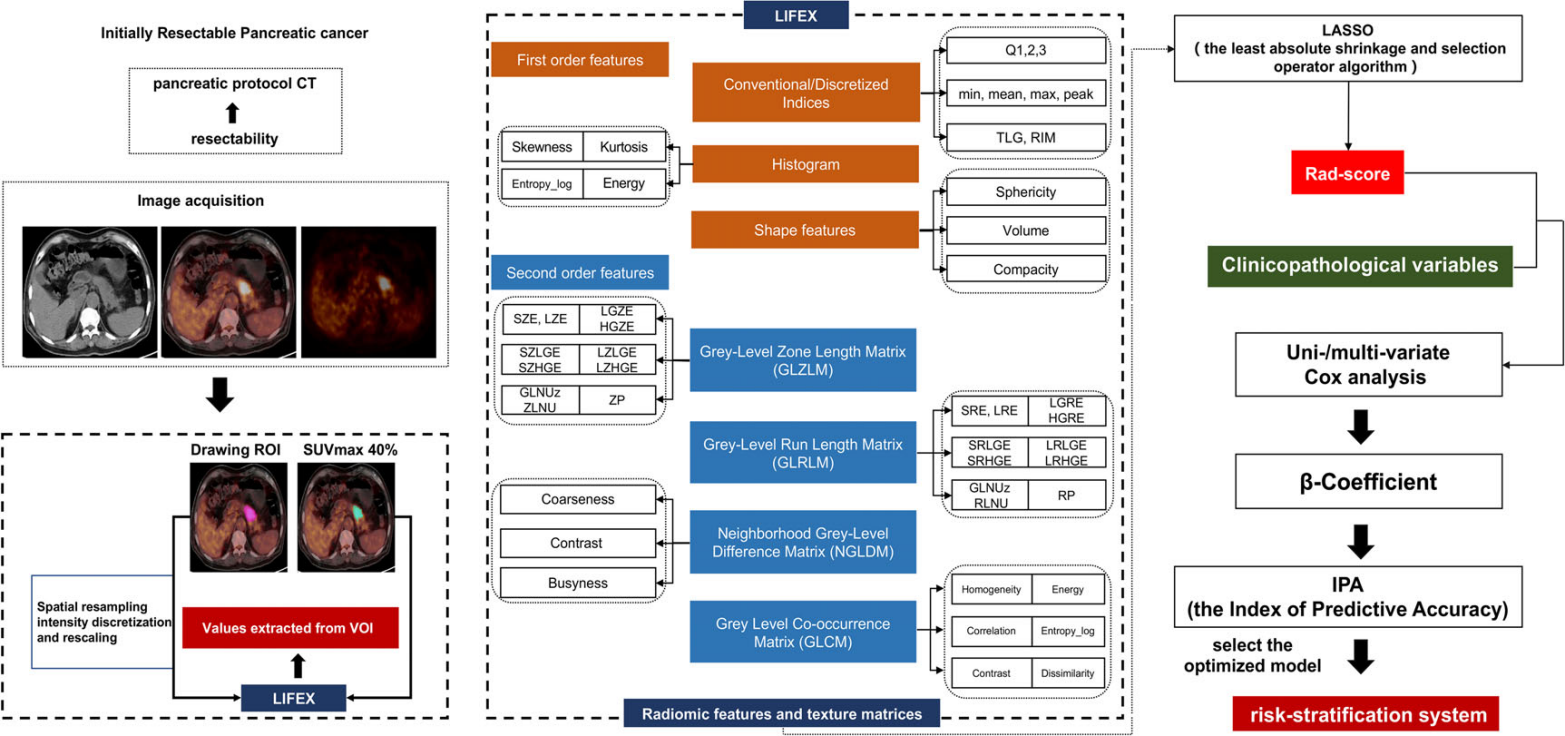
Flowchart of patient selection, radiomic features extraction and stratification system construction.

To extract the PET/CT imaging texture features of the lesions, we applied LIFEx software (v4.00, http://www.lifexsoft.org). PET and CT images in the DICOM format were consecutively imported into LIFEx and automatically fused by the software. Areas with abnormal uptake of ^18^F-FDG on PET and abnormal density on CT were defined as lesions. The final VOI of the primary tumor lesion was automatically defined on PET images with a threshold of 40% of the SUVmax. We performed VOI placement for attenuation correction of low-dose CT images from the PET/CT scan. Then, the spatial resampling was implemented as 2 mm in spacing X, Y, and Z on both PET and CT images by three-dimensional Lagrangian polygon interpolation for all 156 patients. Using a threshold of 40% of the SUVmax, two experienced PET/CT diagnostic physicians semiautomatically delineated the VOI of the target lesion. Texture features were calculated only for VOIs of ≥64 voxels because textural features cannot be accurately quantified for small regions. The PET and CT features were then automatically extracted from the same VOI, and 94 radiomic features were extracted using LIFEx software. Four gray-level matrices were calculated in three dimensions, giving 46 radiomics features (including first-order and second-order features and volumes) for each of the CT-tumor VOIs and 48 radiomics features for each of the PET-tumor VOIs. The radiomic feature extraction process is shown in **Figure 1**. All 94 features are shown in the supplementary material (**Table S1**). Particularly, the forty-eight conventional PET parameters and radiomics features were extracted and in agreement with the IBSI description, including:

- Eight conventional PET parameters: SUVmax, SUVmean, SUVmin, SUVpeak, SUVstd, SUVSkewness, SUVKurtosis, and TLG
- Six descriptors of the image intensity histogram: HISTO_Skewness (asymmetry), HISTO_Kurtosis (flatness), HISTO_ExcessKurtosis, HISTO_Energy (uniformity), HISTO_Entropy_log2, and_log10 (randomness);
- Two shape-based features, that describe shape and compact of VOI: SHAPE_Sphericity, and SHAPE_Compacity;
- Thirty-two textural features: (a) seven features from gray-level co-occurrence matrix (GLCM): describing the correlation between pair of voxels in 13 directions of a three-dimensional space; (b) eleven features from gray-level run length matrix (GLRLM): describing the number and length of run with a certain level of gray in 13 directions of a three-dimensional space; (c) eleven features from gray-level zone length matrix (GLZLM): describing the number and size of zone with a certain level of gray in 13 directions of a three-dimensional space; (d) three features from neighborhood gray-level different matrix (NGLDM): describing the difference between a voxel and its connected neighbors.

46 features from CT including:

- Six conventional CT parameters: HUmax, HUmean, HUmin, HUstd, HUSkewness, and HUKurtosis
- Six descriptors of the image intensity histogram: HISTO_Skewness, HISTO_Kurtosis, HISTO_ExcessKurtosis, HISTO_Energy, HISTO_Entropy_log2, and_log10;
- Two shape-based features: SHAPE_Sphericity, and SHAPE_Compacity;
- Thirty-two textural features that consistent with PET features.

All the texture features were summarized and defined in detail in the **Supplemental Materials**.

### Radiomic Feature Screening and Score Model Establishment

The optimum features were selected from the radiomic features to establish a radiomic model in the training cohort. Next, we used the least absolute shrinkage and selection operator (LASSO) algorithm to screen the optimal predictive features among 94 radiomic features in the development cohort. The radiomic signature score (rad-score) was calculated according to the selected radiomic features of each patient. In this study, we employed the receiver operating characteristic (ROC) and the area under the curve (AUC) to assess the performance of the model in the development set that was verified in the validation set.

### Patient Clinicopathological Characteristics, Treatment Variables and Follow-up

The relevant patient clinicopathological factors included age, sex, body mass index (BMI), the preoperative neutrophil-to-lymphocyte ratio (NLR), the platelet-to-lymphocyte ratio (PLR), and the serum CA19-9 level. The perioperative complications within 90 days of surgery were graded using the Clavien-Dindo classification. The data on the tumor size, differentiation grade, lymph node metastasis, lymphovascular invasion (LVI), perineural invasion (PNI), Ki-67 index and SMAD4 expression by immunohistochemistry were collected. Postoperative data including the timing, regimen and cycles of adjuvant chemotherapy were collected. Pathological T and N stages were classified according to the American Joint Committee on Cancer (AJCC) staging guidelines 8^th^ edition. All the patients had undergone clinical follow-up that included imaging studies (contrast-enhanced CT were required) and blood tests. Clinical evidence of no recurrent disease comprised negative findings on regular imaging and no incremental increase in tumor markers. Recurrence-free survival (RFS) included any recurrence (local or regional, or distant) and death due to any cause.

### Statistical Analysis

Statistical analyses were implemented in R software (version 3.4.3; http://www.R-project.org, R Foundation for Statistical Computing, Vienna, Austria) and SPSS Statistics (version 23.0; IBM, Armonk, NY, USA). To compare continuous variables, we adopted independent samples t or the Mann–Whitney U test, while chi-squared test was used to compare categorical variables. The LASSO algorithm was applied to further identify the optimal features and build the radiomic features score (rad-score) (19–21). A Cox proportional hazards regression model was employed to recognize independent recurrence predictors and calculate hazard ratios (HRs), 95% confidence intervals (CIs), and *β* regression coefficients. Variables that were statistically significant in univariate analysis were included in multivariate analysis and the risk stratification system (P < 0.05). Inspired by Sullivan et al. (22), the *β*-coefficients of each covariate from the Cox proportional hazards regression model were used to generate an integer-based point scoring system for each covariate; the overall score was calculated as the sum of the covariate weighted scores. By dividing the *β* coefficients with the constant values of the largest *β* coefficient in the final model, scores were assigned and multiplied by 10 and rounded to the nearest integer. To select an optimized model, the index of predictive accuracy (IPA) was employed and realized by the IPA function in the “risk regression” package in R (23). As a new bio-informatics tool for biomarker assessment and outcome-based cut-point optimization, X-Tile plot provides a single, global assessment of every possible way of dividing a population into low-, medium-, and high-level marker expression. In our study, X-tile plot was used to generate the optimum cutoff point of risk score according to the highest χ2-value defined by Kaplan–Meier survival analysis and log-rank test (**Figure S1**). X-tile data are presented in a right triangular grid where each point represents a different cut-point. The intensity of the color of each cutoff point represents the strength of the association. The values of risk score were used as input for the X-Tile plots. The groups were divided into low-, intermediate-, and high-risk groups according to cutoff values of risk score (24). The survival of RFS was estimated using the Kaplan–Meier method and compared using the log-rank test. A P value <0.05 was considered statistically significant.

## Results

### Baseline Clinical Characteristics of the Patients

Of the 156 enrolled resected pancreatic cancer patients, 92 were male, accounting for nearly 60%; the mean age was 61.54 ± 8.37 years. The overall median postsurgical follow-up period was 24.8 months (range, 3–84.8). All the resected patients received adjuvant chemotherapy, and 106 (67.5%) patients had recurrence. Regarding tumor pathology, most of the patients had moderate to low differentiation, with high differentiation in 22 patients (14.1%). A total of 52.6% of patients presented with positive lymph node metastases. Additionally, 42.9% of pancreatic cancer patients had a CA19-9 reduction ≥80% compared with the preoperative levels. Positive surgical margins were found in 32 patients (20.5%), and SMAD4 immunohistochemical staining was confirmed as positive in 61 patients (39.1%). PNI and adjuvant therapy regimens were significantly different between the training and validation sets (*p* = 0.038 and *p* = 0.042, respectively). The other clinical features were not significantly different between the groups. Regarding the recurrence pattern of RPC, the most frequent site of recurrence following pancreatectomy for PDAC was the liver. Most patients had recurrence at distant sites, either isolated or multiple sites (n = 91; 58.3%), while liver-only recurrence was found in 30 patients (19.2%), and isolated local recurrence in 15 patients (9.6%). The details of the baseline data are summarized in **Table 1**.

**Table 1:** Clinical characteristics of resectable pancreatic cancer patients at baseline in the training and validation cohorts.

Variables		Overall (N = 156)	Training cohort (N = 109)	Validation cohort (N = 47)	P
Sex					1
	Female	64 (41.0%)	45 (41.3%)	19 (40.4%)	
	Male	92 (59.0%)	64 (58.7%)	28 (59.6%)	
Age (years)					0.527
	>60	92 (59.0%)	62 (56.9%)	30 (63.8%)	
	≤60	64 (41.0%)	47 (43.1%)	17 (36.2%)	
BMI (kg/m^2^)					0.604
	<18.5	15 (9.6%)	10 (9.2%)	5 (10.6%)	
	18.5–24.9	102 (65.4%)	75 (68.8%)	27 (57.4%)	
	25–28	28 (17.9%)	17 (15.6%)	11 (23.4%)	
	28–32	10 (6.4%)	6 (5.5%)	4 (8.5%)	
	>32	1 (0.6%)	1 (0.9%)	0 (0.0%)	
Operation					0.942
	DP	76 (48.7%)	54 (49.5%)	22 (46.8%)	
	PD	71 (45.5%)	49 (45.0%)	22 (46.8%)	
	TP	9 (5.8%)	6 (5.5%)	3 (6.4%)	
NLR					0.562
	≥5	15 (9.6%)	9 (8.3%)	6 (12. 8%)	
	<5	141 (90.4%)	100 (91.7%)	41 (87.2%)	
PLR					0.650
	≥110	114 (73.1%)	78 (71.6%)	36 (76.6%)	
	<110	42 (26.9%)	31 (28.4%)	11 (23.4%)	
ΔCA19-9 decrease					0.128
	<80%	89 (57.1%)	67 (61.5%)	22 (46.8%)	
	≥80%	67 (42.9%)	42 (38.5%)	25 (53.2%)	
R status					0.622
	R0	124 (79.5%)	85 (78.0%)	39 (83.0%)	
	R1	32 (20.5%)	24 (22.0%)	8 (17.0%)	
Tumor differentiation					0.667
	High	22 (14.1%)	14 (12.8%)	4 (8.5%)	
	Median	95 (60.9%)	74 (67.9%)	32 (69.1%)	
	Low	39 (25.0%)	21 (19.3%)	11 (23.4%)	
T stage					0.451
	1	25 (16.0%)	15 (13.8%)	10 (21.3%)	
	2	89 (57.1%)	65 (59.6%)	24 (51.1%)	
	3	42 (26.9%)	29 (26.6%)	13 (27.7%)	
N stage					0.786
	0	74 (47.4%)	56 (51.4%)	26 (55.3%)	
	1	56 (35.9%)	41 (37.6%)	15 (31.9%)	
	2	26 (16.7%)	12 (11.0%)	6 (12.8%)	
LVI					1
	Negative	96 (61.5%)	67 (61.5%)	29 (61.7%)	
	Positive	60 (38.5%)	42 (38.5%)	18 (38.3%)	
PNI					0.038
	Negative	22 (14.1%)	20 (18.3%)	2 (4.3%)	
	Positive	134 (85.9%)	89 (81.7%)	45 (95.7%)	
Ki-67					0.539
	≥50%	132 (84.6%)	94 (86.2%)	38 (80.9%)	
	<50%	24 (15.4%)	15 (13.8%)	9 (19.1%)	
SMAD4 expression					0.448
	Negative	95 (60.9%)	69 (63.3%)	26 (55.3%)	
	Positive	61 (39.1%)	40 (36.7%)	21 (44.7%)	
Clavien-Dindo classification					0.476
	0	108 (69.2%)	73 (67.0%)	35 (74.5%)	
	I–II	46 (29.5%)	35(32.1%)	11 (23.4%)	
	III–V	2 (1.3%)	1(0.9%)	1 (2.1%)	
Adjuvant therapy regimen					0.042
	Gemcitabine-based	89 (57.1%)	62 (56.9%)	27 (57.4%)	
	5FU-based	53 (34.0%)	41 (37.6%)	12 (25.5%)	
	Combining	14 (8.9%)	6 (5.5%)	8 (17.0%)	
Duration of adjuvant therapy					0.544
	<two cycles	53 (34.0%)	36 (33.0%)	17 (36.2%)	
	two to four cycles	40 (25.6%)	26 (23.9%)	14 (29.8%)	
	four to six cycles	63 (40.4%)	47 (43.1%)	16 (34.0%)	
Recurrence site					0.257
	Local	15 (9.6%)	12 (11.0%)	3 (6.4%)	
	Liver	30 (19.2%)	24 (22.0%)	6 (12.8%)	
	Peritoneum	11 (7.1%)	7 (6.4%)	4 (8.5%)	
	Multiple sites	27 (17.3%)	21 (19.3%)	6 (12.8%)	
	Other or uncertain sites	23 (14.7%)	16 (14.7%)	7 (14.9%)	
	None	50 (32.1%)	29 (26.6%)	21 (44.7%)	
Rad-score					0.591
	High	50 (32.1%)	33 (30.3%)	17 (36.2%)	
	Low	106 (67.9%)	76 (69.7%)	30 (63.8%)	
Total risk score					0.214
	High	29 (18.6%)	18 (16.5%)	11 (23.4%)	
	Intermediate	44 (28.2%)	28 (25.7%)	16 (34.0%)	
	Low	83 (53.2%)	63 (57.8%)	20 (42.6%)	

BMI, body mass index; DP, distal pancreatectomy; PD, pancreatoduodenectomy; TP, total pancreatectomy; NLR, neutrophil-to-lymphocyte ratio; PLR, platelet-to-lymphocyte ratio; LVI, lymphovascular invasion; PNI, perineural invasion.

### Feature Extraction and Construction of the Rad-Score

**Figure 2:**
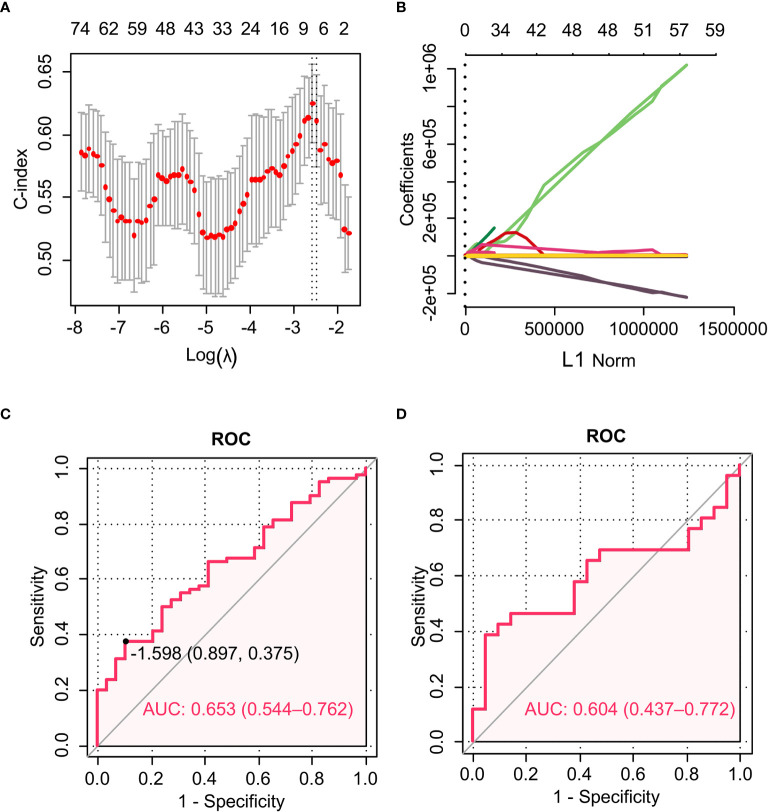
The LASSO algorithm and 10-fold cross-validation were applied to extract the optimal subset of radiomic features. ROC curves for the radiomic model in predicting RFS. **(A)** The AUC reached the peak corresponding to the optimal number of radiomic features when the ln (*λ*) value increased to 0.0925. Optimal features were determined by the AUC value. **(B)** LASSO coefficient profiles of the 94 radiomic features. The vertical line was drawn at the value determined by 10-fold cross-validation, where the optimal *λ* generated six non-zero coefficients. **(C)** ROC curve of the training cohort. **(D)** ROC curve of the validation cohort.

Using the LASSO algorithm and 10-fold cross-validation, we extracted the optimal subset of radiomic features. The value of 0.0925 was determined as the optimal *λ*, and eventually six of 94 radiomic features with non-zero coefficients were chosen in the training set (**Figures 2A, B**). The six selected radiomic features were PET*GLZLM_LZE_*, PET*SHAPE_Sphericity_*, PET*CONVENTIONAL_SUVbwSkewness_*, CT*SHAPE_Sphericity_*, CT*SHAPE_Compacity_*, and CT*CONVENTIONAL_HUSkewness_*. Among the six features, the first three were related to PET, while the remaining three features were derived from CT imaging. To calculate the rad-score of each patient, we constructed a logistic regression formula containing the above selected features as follows: rad-score = PET*GLZLM_LZE_* × 7.84 × 10^-6^–PET*SHAPE_Sphericity_* × 1.354944481 – PET*CONVENTIONAL_SUVbwSkewness_* × 0.219952725–CT*CONVENTIONAL_HUSkewness_* ×0.078539806-CT*SHAPE_Sphericity_*×0.629610997-CT*SHAPE_Compacity_* × 0.073882711. In this formula, element (i, j) of GLZLM corresponds to the number of homogeneous zones of j voxels with the intensity i in an image and is called GLZLM(i, j) thereafter. $GLZLM\_LZE=\frac{1}{H}\Sigma_{i}\Sigma_{j}GLZLM(i,j)\cdot j^{2}$, where H corresponds to the number of homogeneous zones in the VOI. $SHAPE\_Sphericity=\frac{\pi^{1/3}\cdot {\left(6V\right)}^{2/3}}{A},SHAPE\_Compacity=\frac{A^{3/2}}{V}\;,$ Where V and A correspond to the volume and the surface of the VOI based on the Delaunay triangulation. $CONVENTIONAL\_Skewness=\frac{\frac{1}{N\;}\Sigma_{i}i\left(I\left(i\right)-\bar{I}\right){\;}^{3}}{{\left(\sqrt{\frac{1}{N\;}\Sigma_{i}\left(I\left(i\right)-\bar{I}\right){\;}^{2}}\right)}^{3}},$ where *I(i) *corresponds to the number of voxels with intensity *i*, *N* the total number of voxels in the VOI and $\bar{I}$ the average of gray-levels.

**Figure 3:**
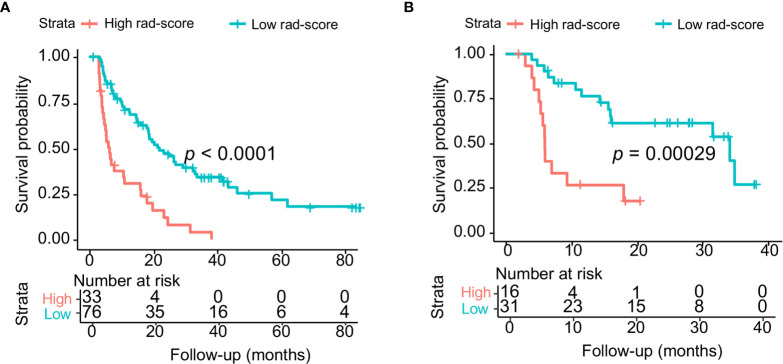
**(A)** Kaplan–Meier survival curves for the high and low rad-score groups in the training cohort. **(B)** Kaplan–Meier survival curves for the high and low rad-score groups in the validation cohort.

To evaluate the performance of the selected radiomic features in predicting the RFS of RPC patients, ROC curves were used. The rad-score had AUCs of 0.653 (95% CI, 0.544–0.762) in the training set and 0.604 (95% CI, 0.437–0.772) in the validation cohort (**Figures 2C, D** **)**. The cutoff value of the rad-score was −1.598. The C-indexes of the difference in the probability of survival between the high and low rad-score groups in the training and validation cohorts were 0.784 (95% CI, 0.693–0.875) and 0.836 (95% CI, 0.713–0.959), respectively. Kaplan-Meier survival curves for the high and low rad-score groups in the training and validation cohort were depicted (**Figures 3A, B** **)**. Waterfall plots were drawn to display the rad-score of each patient (**Figure S2A**).

### Development and Validation of a Risk Stratification System for Recurrence Prediction

Each clinicopathological factor was converted into a categorical variable, and then we performed univariate and multivariable Cox analyses to determine the independent risk factors for recurrence in PDAC patients who had undergone surgery. Variables with P <0.05 in the univariate analysis were further incorporated into multivariable Cox analysis including the following factors: rad-score (high *vs* low), tumor differentiation (low *vs* moderate-high), lymph node metastasis (positive *vs* negative), ΔCA19-9 (<80% decrease *vs* ≥80% decrease), LVI (positive *vs* negative), SMAD4 expression (negative *vs* positive) and duration of adjuvant therapy (<two *vs* two to four *vs* four to six cycles). The results of multivariable Cox analysis revealed that the rad-score, ΔCA19-9 and duration of adjuvant therapy were three independent risk factors for recurrence in PDAC patients who had undergone resection [rad-score: HR, 2.891, (95% CI, 1.704–4.905; P < 0.001); ΔCA19-9: HR, 1.842, (95% CI, 1.088–3.118; P = 0.023); and duration of adjuvant therapy: two to four cycles HR, 1.703, (95% CI, 0.904–3.206), <two cycles HR, 2.388, (95% CI, 1.161–4.909; P<0.001)] (**Table 2**). Independent risk factors were selected to establish a recurrence prediction model, the risk stratification system. The score was calculated according to the six prognostic predictors weighted by *β* regression coefficients in multivariable Cox analysis, which were rounded into integer values using the method by Sullivan et al. (22): 11 points for a high rad-score, nine points for four to six cycles of adjuvant chemotherapy, seven points for low differentiation, six points for ≥80% decrease in ΔCA19-9, five points for <two cycles of adjuvant chemotherapy, three points for positive LVI, three points for lymph node metastasis, and one point for positive SMAD4 expression. The total risk score ranged from 0 to 40 points. According to X-tile analysis, 16 and 29 points were used as the cutoff values, 64 (58.72%), 34 (31.19%), and 11 patients (10.09%) were categorized into the low- (0 to 16), intermediate- (17 to 29), and high-risk (30 to 40) groups, respectively. The results of the validation cohort were similar: there were 51.1, 31.9, and 17.0% of the patients in the low-, medium-, and high-risk group, respectively. Waterfall plots were drawn according to the risk score (**Figure S2B**).

**Table 2:** Construction of a risk-stratification system for risk stratification in RPC patients.

Variables	Univariate analysis	Multivariate analysis	HR (95% CI)	β-Coefficient	Score assigned
	P	P			
Rad-score	<0.001	<0.001			
Low			Reference group	.	0
High			2.891 (1.704–4.905)	1.062	11
Differentiation	<0.001	0.065			
Median-high			Reference group	.	0
Low			2.103 (0.955–4.63)	0.743	7
Lymph node metastasis	0.001	0.3			
Negative			Reference group	.	0
Positive			1.312 (0.785–2.193)	0.272	3
ΔCA19-9 decrease	0.001	0.023			
≥ 80%			Reference group	.	0
< 80%			1.842 (1.088-3.118)	0.611	6
LVI	0.001	0.302			
Negative			Reference group	.	0
Positive			1.296 (0.792–2.12)	0.259	3
SMAD4 expression	0.036	0.633			
Positive			Reference group	.	0
Negative			1.145 (0.657–1.997)	0.136	1
Adjuvant chemotherapy		< 0.001			
four to six cycles			Reference group	.	0
two to four cycles	0.0116	0.099	1.703 (0.904–3.206)	0.532	5
<two cycles	<0.001	0.018	2.388 (1.161–4.909)	0.87	9

LVI, lymphovascular invasion.

To predict the one-year recurrence risk, the Brier scores of the final model (rad-score + ΔCA19-9 ≥80% decrease + tumor differentiation + lymph node metastasis + LVI + SMAD4 expression + adjuvant chemotherapy) in the training and validation cohorts were 0.14 and 0.20, which corresponded to IPA values of 30.6 and 20.6%, respectively. **Table 3** indicates the changes in the Brier score and IPA at 1 year regarding RFS when each of the seven variables was excluded from the model, suggesting that the incorporation of these seven variables in the final model was favorable to optimize the prediction accuracy of the risk stratification system.

**Table 3:** Comparison of the different models regarding RFS in the training and validation cohorts.

No. of Variables	Variables included in the model	Brier score	IPA at 1year, %	IPA drop
Training cohort				
1 (final model)	Rad-score + ΔCA19-9 + Differentiation + Lymph node metastasis + LVI + SMAD4 + Adjuvant therapy	0.135551151	0.305826195	0
2	ΔCA19-9 + Differentiation + Lymph node metastasis + LVI + SMAD4 + Adjuvant therapy	0.139392659	0.286153371	0.019672824
3	Rad-score + ΔCA19-9 + Lymph node metastasis + LVI + SMAD4 + Adjuvant therapy	0.146757759	0.248435803	0.057390392
4	Rad-score + ΔCA19-9 + Differentiation + LVI + SMAD4 + Adjuvant therapy	0.136588037	0.300516179	0.005310016
5	Rad-score + Differentiation + Lymph node metastasis + LVI + SMAD4 + Adjuvant therapy	0.144486321	0.260068112	0.045758083
6	Rad-score + ΔCA19-9 + Differentiation + Lymph node metastasis + SMAD4 + Adjuvant therapy	0.143317882	0.26605183	0.039774365
7	Rad-score + ΔCA19-9 + Differentiation + Lymph node metastasis + LVI + Adjuvant therapy	0.14245439	0.270473875	0.03535232
8	Rad-score + ΔCA19-9 + Differentiation + Lymph node metastasis + LVI	0.138483372	0.290809936	0.015016259
Validation cohort				
1 (final model)	Rad-score + ΔCA19-9 + Differentiation + Lymph node metastasis + LVI + SMAD4 + Adjuvant therapy	0.196208964	0.206180219	0
2	ΔCA19-9 + Differentiation + Lymph node metastasis + LVI + SMAD4 + Adjuvant therapy	0.19784561	0.199558697	0.006621522
3	Rad-score + ΔCA19-9 + Lymph node metastasis + LVI + SMAD4 + Adjuvant therapy	0.198989105	0.194932359	0.01124786
4	Rad-score + ΔCA19-9 + Differentiation + LVI + SMAD4 + Adjuvant therapy	0.19977719	0.191743931	0.014436288
5	Rad-score + Differentiation + Lymph node metastasis + LVI + SMAD4 + Adjuvant therapy	0.222610612	0.099364758	0.106815461
6	Rad-score + ΔCA19-9 + Differentiation + Lymph node metastasis + SMAD4 + Adjuvant therapy	0.200846238	0.187418792	0.018761427
7	Rad-score + ΔCA19-9 + Differentiation + Lymph node metastasis + Adjuvant therapy	0.19950763	0.192834516	0.013345703
8	Rad-score + ΔCA19-9 + Differentiation + Lymph node metastasis + LVI	0.200743146	0.187835881	0.018344337

ΔCA19-9 indicates a level decrease ≥80%; SMAD4 indicates a negative status; and the duration of adjuvant therapy was divided into three categories: ＜two cycles, two to four cycles, and four to six cycles.

**Figure 4:**
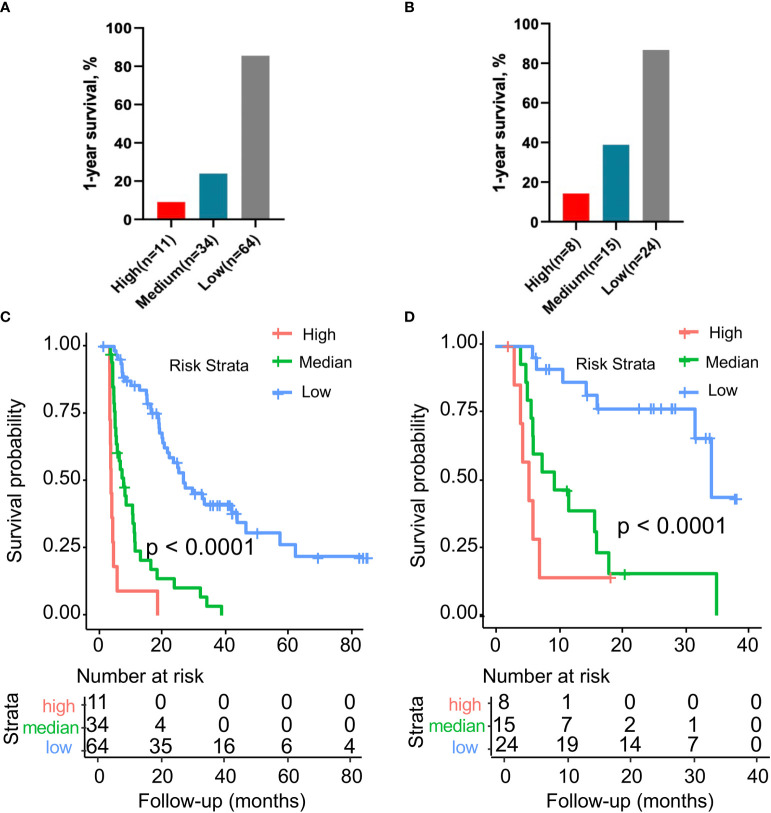
**(A)** Comparison of the 1-year recurrence-free survival rate in the training cohort with different risk stratifications. **(B)** Comparison of the 1-year recurrence-free survival rate in the validation cohort with different risk stratifications. **(C)** Kaplan–Meier survival curves for the three risk stratification groups in the training cohort. **(D)** Kaplan–Meier survival curves for the three risk stratification groups in the validation cohort. Risk stratification system: low risk, 0 to 16 points; medium risk, 17 to 29 points; high risk, 30 to 40 points.

The prediction accuracy of this risk stratification system for PDAC recurrence, evaluated by the C-index, was 0.890 (95% CI, 0.835–0.945) for the training cohort and 0.865 (95% CI, 0.778–0.952) for the validation cohort. The 1-year RFS rates of the low-, intermediate-, and high-risk groups were 85.5, 24.0, and 9.1%, respectively (**Figure 4A**). In the validation cohort, the 1-year survival rates in the low-, intermediate-, and high-risk groups were 86.7, 38.9, and 14.3%, respectively (**Figure 4B**). In this study, the median follow-up time was 24.8 months (range, 3 to 84.8 months), and survival data were obtained to explore the prognostic value of risk stratification. In the training cohort, patients in the high-risk group (n = 11) showed significantly shorter survival times than those in the low-risk (n = 64) and intermediate-risk groups (n = 34) (P < 0.0001; **Figure 4C**). A similar trend was observed in the validation cohort risk group (P < 0.0001; **Figure 4D**).

## Discussion

This study presents an internally validated risk stratification system for predicting early relapse in patients with resected pancreatic cancer. Integrating preoperative ^18^F-PET/CT radiomic features, changes in a sensitive biomarker (ΔCA19-9), pathological characteristics (tumor differentiation, lymph node metastasis, LVI, and SMAD4 expression), and the duration of adjuvant chemotherapy associated with early recurrence, the patients were scored ranging from 0 to 40. Herein, we classified patients into three groups with corresponding points assigned as follows: low-risk group (0–16 points), intermediate-risk group (17–29 points), and high-risk group (30–40 points). Compared with low-risk patients, the relapse rate was two to three times higher in the intermediate-risk group and six to nine times higher in the high-risk group. Our risk stratification system was confirmed to stratify patients by increasing risk of recurrence accurately in an independent internally validation cohort. Accordingly, we recommend that patients in the high- and intermediate-risk groups be followed-up for recurrence at regular intervals, such as every 2–3 months.

The six selected radiomic features were used to construct a formula of rad-score calculation. The *GLZLM* provides information on the size of homogeneous zones for each gray-level in three dimensions. From this matrix, 11 texture indices are computed. More precisely, *GLZLM is* particularly efficient to characterize the texture homogeneity, which had provided better characterizations than GLRLM or GLCM for the classification of textures in PET images. *SHAPE_Sphericity* and *SHAPE_Compacity* reflect how spherical and compact the VOI is. Sphericity is a measure of the roundness of the shape of the tumor region relative to a sphere and is equal to 1 for a perfect sphere. *CONVENTIONAL_Skewness* measures the asymmetry of the gray-level distribution. Depending on where the tail is elongated and the mass of the distribution is concentrated, this value can be positive or negative. Fiorino et al. (25) established a radiomic-based index with good performance derived from ^18^F-FDG PET/CT radiomics features to predict distant RFS in 176 patients with locally advanced pancreatic cancer treated with radio-chemotherapy. In their study, one-hundred-ninety-eight radiomic features were extracted, and only two robust features were included, Morpholoigical_COMshift and Statistical_P_10_. Specifically, the COMshift is the distance between the VOI centroid and the intensity-weighted VOI centroid. P_10_ is an intensity-based statistical feature which represents the 10^th^ percentile of the set of gray levels of the voxels included in the VOI. Exploration of CT-based radiomics to predict survival prognosis or treatment response in pancreatic cancer is more common (14, 17, 18). Researcher also developed a multiparametric MRI radiomic nomogram for preoperative evaluation of early recurrence risks in resectable pancreatic cancer, incorporating the radiomic signatures from T1-w, T2-w, portal venous phase, and arterial phase and clinical parameters (26). Recently, radiogenomics is and emerging field that integrates “radiomics” and “genomics”, which may aid in the development of precision medicine. Iwatate et al. (27) found that radiogenomics could predict p53 mutations and in turn the prognosis of PDAC patients. Pancreatic cancer remains one of the most lethal malignancies, radiomics may have the potential to address some problems but further validation in larger-scale, multicenter studies and in randomized control trials is required.

In PDAC, the median survival of surgery-only patients is 15–20 months, and the 5-year survival rate is 8–15% (28–31). Even after curative resection, 69–75% of patients with pancreatic cancer show recurrence within 2 years (28–30, 32, 33). Clinicopathological parameters, including tumor differentiation, lymph node metastasis, and LVI, were found to be strong predictors of prognosis in PDAC patients who had undergone surgical resection (3, 34–36). The NLR and PLR are inflammatory indicators that are correlated with OS in PADC patients (21, 37–39). The Ki-67 proliferative index could be used in the survival prediction of resectable PDAC, and an index above 50% was negatively related to survival compared with other patients (20). However, the above indices showed no predictive value for recurrence in our study.

CA19-9 is the most studied efficacy predictor and prognostic biomarker in PDAC (40, 41). Patients with preoperative CA19-9 ≥1,000 U/ml generally showed a poor surgical benefit; however, subgroups with CA19-9 levels decreased postoperatively may still achieve a survival benefit. For borderline or locally advanced PDAC patients, the CA19-9 response (reduction >50%) during primary or neoadjuvant chemotherapy may provide insight into a patient selection that will benefit from surgical resection (42–44). However, Tsai et al. (45) retrospectively analyzed 131 PDAC patients and suggested that following neoadjuvant therapy, CA19-9 normalization is a robust prognostic factor for longer survival. In this study, we found that a reduction in the CA19-9 levels of ≥80% after resection indicated a better prognosis. SMAD4 is a widely known tumor suppressor that is inactivated in more than half of PDAC patients (46). SMAD4 deficiency induced by genomic deletions or truncated mutations are associated with an inferior prognosis in pancreatic cancer (47). Our results demonstrated a SMAD4 negative status is independently significantly correlated with RFS in PDAC patients.

The high relapse rate of pancreatic cancer following curative surgery suggests the necessity of adjuvant therapy. Currently, the regimens are based on 5-FU or gemcitabine, and a combination chemotherapy with gemcitabine and capecitabine or mFOLFIRINOX has been verified to improve OS (48, 49). We retrospectively analyzed the duration of adjuvant treatment grouped into three categories (without or <2 months, 2–4 months, and 4–6 months) and found that a longer duration conferred benefit to RPC patients, demonstrating the adequate adjuvant course for the high recurrence risk population, and may present a potentially promising treatment option of consolidation therapy in pancreatic cancer patients. Most of the patients in our study initiated adjuvant therapy within eight weeks postoperatively according to the current consensus (50), and previous results showed that patients still benefit from adjuvant therapy started more than 12 weeks after surgical resection (51).

From 2013, eligible patients were continuously recruited, and our study may present as one of the largest sample sizes published in pancreatic cancer to date. The follow-up duration ranged from 3 to 84.8 months. Our results showed that the recurrence risk stratification system had good predictive performance. Nevertheless, our study has many limitations. First, selection bias exists as a potential flaw inherent in retrospective analysis. Prospective multi-center studies on ^18^F-FDG PET/CT of resected PDAC are needed to limit the bias and verify whether certain features could work as reliable predictors. Second, our research discussed only the predictive performance of the model for patients undergoing surgical resection, but the intratumoral heterogeneity of pancreatic cancer should be further studied. Third, evidence from ESPAC-4 and PRODIGE24/CCTG PA.6 demonstrated that the adjuvant combination of gemcitabine and capecitabine, or modified FOLFIRINOX regimens, shows longer survival than gemcitabine alone in resected pancreatic cancer patients (31, 49). Nearly all the patients in our study used gemcitabine or 5-FU mono-chemotherapy on account of the time of consultation. Better survival could be achieved when patients received new combination regimen.

## Conclusion

The risk stratification system based on ^18^F-FDG PET/CT radiomic features demonstrated good performance in relapse prediction after pancreatectomy in RPC patients, providing strong recommendations for adequate adjuvant therapy courses, particularly for patients with a high risk of relapse, and maybe a useful method for monitoring and surveillance in a clinical setting.

## Data Availability statement

The original contributions presented in the study are included in the article/**Supplementary Material**. Further inquiries can be directed to the corresponding authors.

## ethics Statement

The studies involving human participants were reviewed and approved by the Ethics Committee of the Fudan University Shanghai Cancer Center. The patients/participants provided their written informed consent to participate in this study.

## Author Contributions

MW, BG, and SS conceptualized and designed the study. SLS, BZ WW, JX, and XY performed analysis. XY and SS interpreted the data. MW and BG drafted the manuscript. XY and SS revised the manuscript. All authors contributed to the article and approved the submitted version.

## Funding

This study was jointly funded by the National Natural Science Foundation of China (No. 81772555, 81802352 and 81902428), the National Science Foundation for Distinguished Young Scholars of China (No. 81625016), the Shanghai Sailing Program (No. 19YF1409400 and 20YF1409000), the Shanghai Rising-Star Program (No. 20QA1402100), the Shanghai Anticancer Association Young Eagle Program (No.SACA-CY19A06), the Clinical and Scientific Innovation Project of Shanghai Hospital Development Center (No. SHDC12018109 and SHDC12019109) the Clinical Research Plan of SHDC (No.SHDC2020CR1006A), and the Scientific Innovation Project of Shanghai Education Committee (No. 2019-01-07-00-07-E00057).

## Conflict of Interest

The authors declare that the research was conducted in the absence of any commercial or financial relationships that could be construed as a potential conflict of interest.
