# Supplementary material for: A Novel Validated Recurrence Stratification System Based on 18F-FDG PET/CT Radiomics to Guide Surveillance After Resection of Pancreatic Cancer
Source: Front Oncol. 2021 May 12;11:650266. doi: 10.3389/fonc.2021.650266 (PMC8149949; doi:10.3389/fonc.2021.650266)
Supplement: Supplementary file 1 [file DataSheet_1.pdf]

Supplementary Material

Table S1. The 94 radiomic features of ^{18}F-FDG PET-CT extracted from LIFEx software.

Figure S1

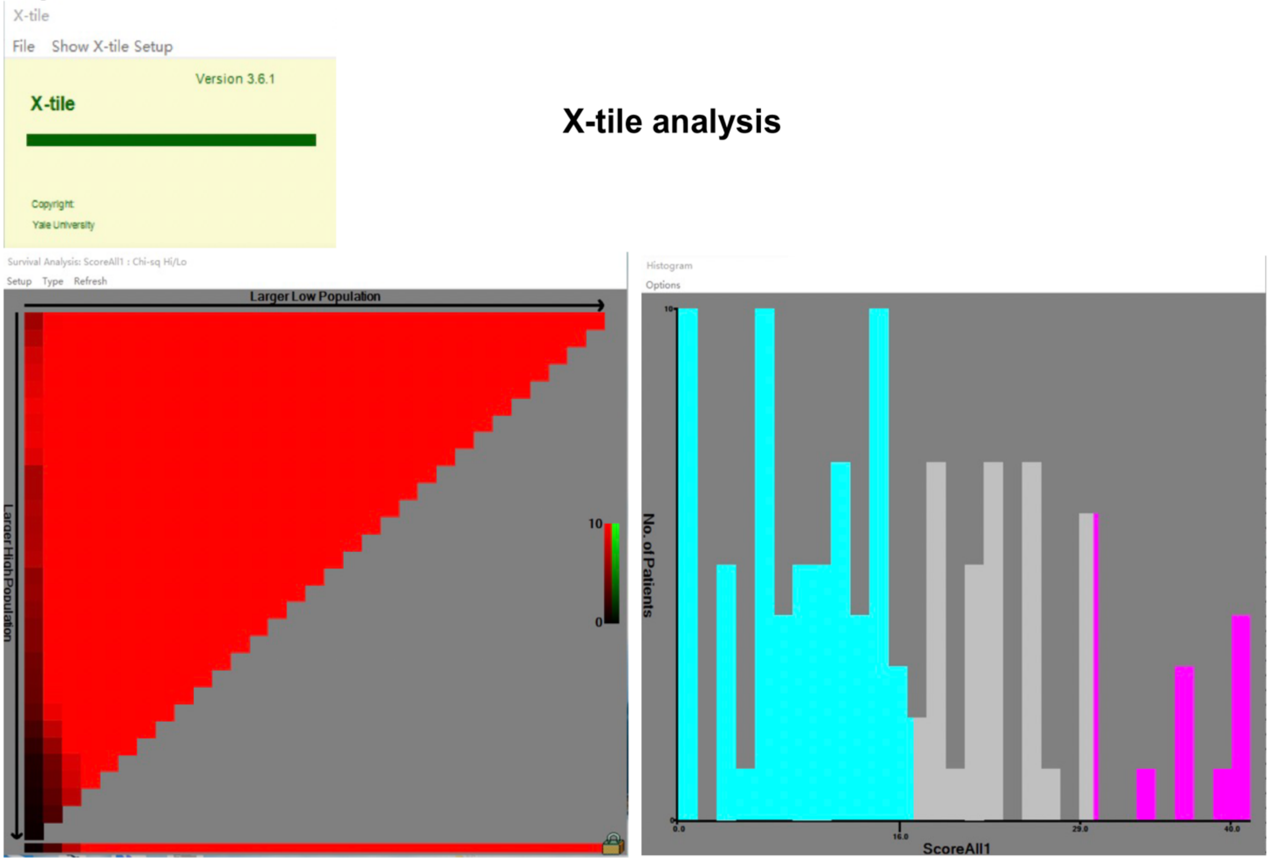

Figure S1. Novel validated recurrence stratification system was divided into low-, intermediate-, and high-risk groups according to cutoff values determined by X-tile plots.

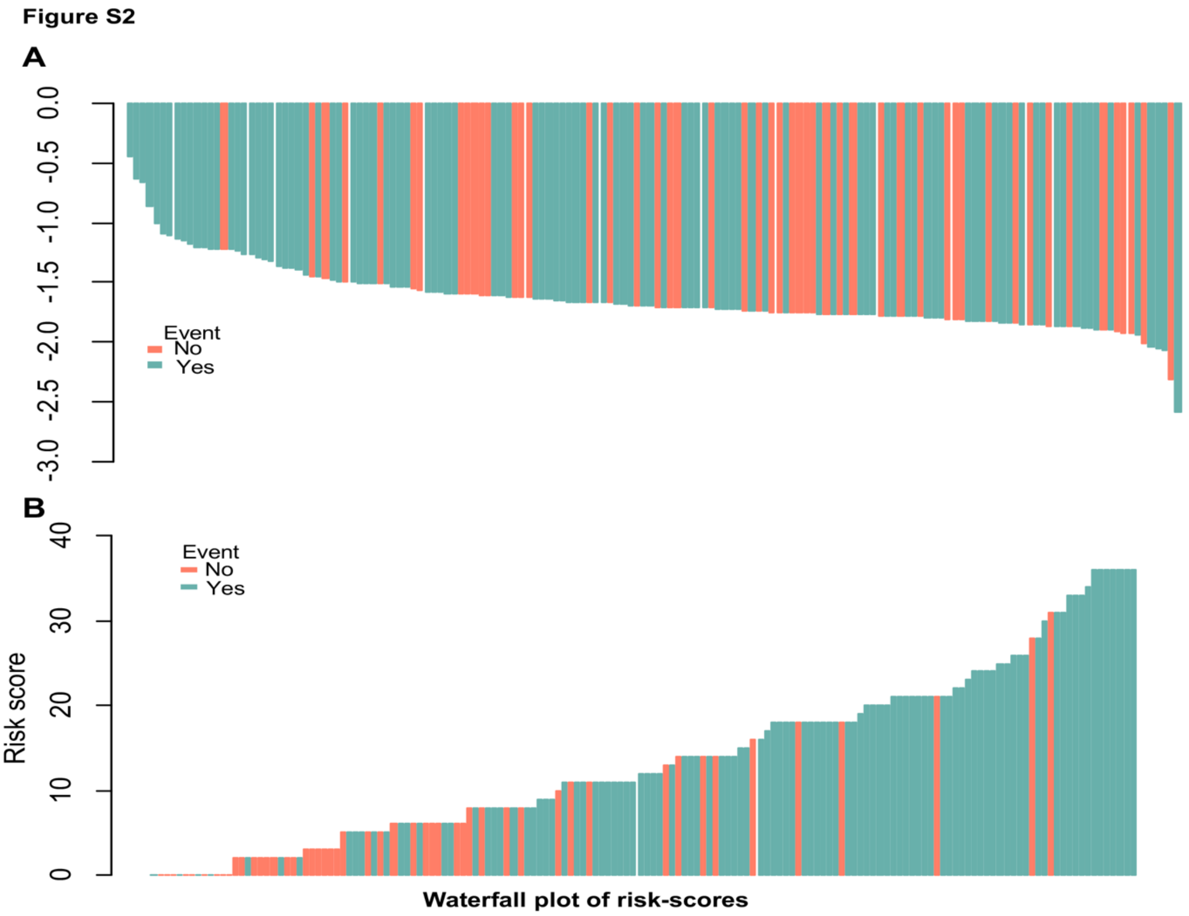

Figure S2.

(A) Waterfall plot were drawn to display the rad-score (score of radiomic features) for every patient.

(B) Waterfall plot according to the risk score for every RPC patient.

Definition of texture features

First order features:

1. Conventional Indices

1.1 *CONV_Q1,Q2,Q3*

A quartile is a type of quantile which divides the number of data points into four more or less equal parts, or quarters. Due to the fact that the data needs to be ordered from smallest to largest in order to compute quartiles, quartiles are a form of Order statistic.

CONVENTIONALQ1: The first quartile (Q1) is defined as the middle number between the smallest number and the median of the data set. It is also known as the lower quartile or the 25th empirical quartile and it marks where 25% of the data is below or to the left of it (if data is ordered on a timeline from smallest to largest).

CONVENTIONALQ2: The second quartile (Q2) is the median of a data set and 50% of the data lies below this point.

CONVENTIONALQ3: The third quartile (Q3) is the middle value between the median and the highest value of the data set. It is also known as the upper quartile or the 75th empirical quartile and 75% of the data lies below this point.

1.2 *CONVmin*, *CONVmean*, *CONVmax, CONVpeak*

CONVmin: reflects the minimum of value (in choosen unit) in the Volume of Interest.

CONVmean: reflects the average value (in choosen unit) in the Volume of Interest.

CONVmax: reflects the maximum value (in choosen unit) in the Volume of Interest.

CONVpeak: reflects the *CONVmean* in a sphere with a volume of ~1 mL and located so that the average value in the VOI is maximum.

1.3 *CONV_TLG(mL), CONV_RIM*

CONV_TLG(mL) is the Total Lesion Glycolysis defined as the product of *SUVmean* by *Volume* in mL.

is the envelope Intensity Mean "*CONV_RIM_mean*" of successive layers of voxels from the outside of *CONV_RIM* is the region to the inside. Each layer is 1 voxel thick. These envelopes are getting smaller and smaller (3D erosion of 1 voxel) up to the center of the ROI.

2. Indices from Histogram

2.1 HISTO_Skewness

is the asymmetry of the grey-level distribution in the histogram.

$$HISTO\_Skewness = \frac{\frac{1}{E}\sum_i (\mathrm{HISTO(i)} - \overline{\mathrm{HISTO}})^3}{\left(\sqrt{\frac{1}{E}\sum_i (\mathrm{HISTO(i)} - \overline{\mathrm{HISTO}})^2}\right)^3}$$

Where *HISTO*(*i*) corresponds to the number of voxels with intensity *i*, *E* the total number of voxels in the Volume of Interest and $\overline{\mathrm{HISTO}}$the average of grey-levels in the histogram.

2.2 HISTO_Kurtosis

reflects the shape of the grey-level distribution (peaked or flat) relative to a normal distribution.

$$HISTO\_Kurtosis = \frac{\frac{1}{E}\sum_i (\mathrm{HISTO(i)} - \overline{\mathrm{HISTO}})^4}{\left(\sqrt{\frac{1}{E}\sum_i (\mathrm{HISTO(i)} - \overline{\mathrm{HISTO}})^2}\right)^2}$$

Where *HISTO*(*i*) corresponds to the number of voxels with intensity *i*, *E* the total number of voxels in the Volume of Interest and $\overline{\mathrm{HISTO}}$the average of grey-levels in the histogram.

2.3 HISTO_Entropy_log10, HISTO_Entropy_log 2

reflects the randomness of the distribution.

$HISTO\_Entropylog10 = -\sum_i \mathrm{p(i)} \cdot \log_{10}(\mathrm{p(i)} + \varepsilon)$

$HISTO\_Entropylog10 = -\sum_i \mathrm{p(i)} \cdot \log_{2}(\mathrm{p(i)} + \varepsilon)$

Where *p*(*i*) is the probability of occurrence of voxels with intensity *i* and *ε*= 2e-16.

2.4 HISTO_Energy
reflects the uniformity of the distribution.
HISTO_Energy=$\sum_i p(i)^2$

3. Indices from shape
3.1 SHAPE_Sphericity
is how spherical a Volume of Interest is. Sphericity is equal to 1 for a perfect sphere.
SHAPE_Sphericity=$\frac{\pi^{1/3} \cdot (6V)^{2/3}}{A}$
Where *V* and *A* correspond to the volume and the surface of the Volume Of Interest based on the Delaunay triangulation.

3.2 SHAPE_Volume (mL and voxels)
is the Volume of Interest in mL and in voxels.
SHAPE_Volume=$\sum_i V_i$
Where *Vi* corresponds to the volume of voxel *i* of the Volume Of Interest.

3.3 SHAPE_Compacity
reflects how compact the Volume of Interest is.
SHAPE_Compacity=$\frac{A^{3/2}}{V}$
Where *V* and *A* correspond to the volume and the surface of the Volume Of Interest based on the Delaunay triangulation.

Second order features:

1. GLZLM
The grey-level zone length matrix (GLZLM) [Thibault] provides information on the size of homogeneous zones for each grey-level in 3 dimensions (or 2D). It is also named Grey Level Size Zone Matrix (GLSZM). From this matrix, 11 texture indices are computed. Element (*i*, *j*) of GLZLM corresponds to the number of homogeneous zones of *j* voxels with the intensity *i* in an image and is called *GLZLM*(*i*, *j*) thereafter.

1.1 GLZLM_SZE, GLZLM_LZE
Short-Zone Emphasis or Long-Zone Emphasis is the distribution of the short or the long homogeneous zones in an image.
GLZLM_SZE=$\frac{1}{H}\sum_i \sum_j \frac{GLZLM(i,j)}{j^2}$
GLZLM_LZE=$\frac{1}{H}\sum_i \sum_j GLZLM(i,j) \cdot j^2$
Where *H* corresponds to the number of homogeneous zones in the Volume of Interest.

1.2 GLZLM_LGZE, GLZLM_HGZE
Low Gray-level Zone Emphasis or High Gray-level Zone Emphasis is the distribution of the low or high grey-level zones
GLZLM_LGZE=$\frac{1}{H}\sum_i \sum_j \frac{GLZLM(i,j)}{i^2}$
GLZLM_HGZE=$\frac{1}{H}\sum_i \sum_j GLZLM(i,j) \cdot i^2$

1.3 GLZLM_SZLGE, GLZLM_SZHGE
Short-Zone Low Gray-level Emphasis or Short-Zone High Gray-level Emphasis is the distribution of the short homogeneous zones with low or high grey-levels.
GLZLM_SZLGE=$\frac{1}{H}\sum_i \sum_j \frac{GLZLM(i,j)}{i^2 \cdot j^2}$
GLZLM_SZHGE=$\frac{1}{H}\sum_i \sum_j \frac{GLZLM(i,j) \cdot i^2}{j^2}$

1.4 GLZLM_LZLGE, GLZLM_LZHGE
Long-Zone Low Gray-level Emphasis or Long-Zone High Gray-level Emphasis is the distribution of the long homogeneous zones with low or high grey-levels.

$GLZLM\_LZLGE = GLZLM\_SZHGE = \frac{1}{H}\sum_i \sum_j \frac{GLZLM(i,j) \cdot j^2}{i^2}$

$GLZLM\_LZHGE = \frac{1}{H}\sum_i \sum_j GLZLM(i,j) \cdot i^2 \cdot j^2$

1.5 GLZLM_GLNUz, GLZLM_ZLNU
Gray-Level Non-Uniformity for zone or Zone Length Non-Uniformity is the nonuniformity of the grey-levels or the length of the homogeneous zones.

$GLZLM\_GLNUz = \left(\frac{1}{H}\sum_i \left(\sum_j GLZLM(i,j)\right)^2\right)$

$GLZLM\_ZLNU = \left(\frac{1}{H}\sum_j (\sum_i GLZLM(i,j))^2\right)$

1.6 GLZLM_ZP
Zone Percentage measures the homogeneity of the homogeneous zones.

$GLZLM\_ZP = \frac{H}{\sum_i \sum_j (j \cdot GLZLM(i,j))}$

2. GLRLM
The grey-level run length matrix (GLRLM) [Xu] gives the size of homogeneous runs for each grey level. This matrix is computed for the 13 different directions in 3D (4 in2D) and for each of the 11 texture indices derived from this matrix, the 3D value is the average over the 13 directions in 3D (4 in 2D). The element (i, j) of GLRLM corresponds to the number of homogeneous runs of j voxels with intensity i in an image and is called $GLRLM(i, j)$ thereafter.

2.1 GLRLM_SRE, GLRLM_LRE
Short-Run Emphasis or Long-Run Emphasis is the distribution of the short or the long homogeneous runs in an image.

$GLRLM\_SRE = \textit{Average over } 13 \textit{ (or } 4\textit{) directions}\left(\frac{1}{H}\sum_i \sum_j \frac{GLRLM(i,j)}{j^2}\right)$

$GLRLM\_LRE = \textit{Average over } 13 \textit{ (or } 4\textit{) directions}\left(\frac{1}{H}\sum_i \sum_j GLRLM(i,j) \cdot j^2\right)$

Where H corresponds to the number of homogeneous runs in the Volume of Interest

2.2 GLRLM_LGRE, GLRLM_HGRE
Low Gray-level Run Emphasis or High Gray-level Run Emphasis is the distribution of the low or high grey-level runs.

$GLRLM\_LGRE = \textit{Average over } 13 \textit{ (or } 4\textit{) directions}\left(\frac{1}{H}\sum_i \sum_j \frac{GLRLM(i,j)}{i^2}\right)$

$GLRLM\_HGRE = \textit{Average over } 13 \textit{ (or } 4\textit{) directions}\left(\frac{1}{H}\sum_i \sum_j GLRLM(i,j) \cdot i^2\right)$

2.3 GLRLM_SRLGE, GLRLM_SRHGE
Short-Run Low Gray-level Emphasis or Short-Run High Gray-level Emphasis is the distribution of the short homogeneous runs with low or high grey-levels.

$GLRLM\_SRLGE = \textit{Average over } 13 \textit{ (or } 4\textit{) directions}\left(\frac{1}{H}\sum_i \sum_j \frac{GLRLM(i,j)}{i^2 \cdot j^2}\right)$

$GLRLM\_SRHGE = \textit{Average over } 13 \textit{ (or } 4\textit{) directions}\left(\frac{1}{H}\sum_i \sum_j \frac{GLRLM(i,j) \cdot i^2}{j^2}\right)$

2.4 GLRLM_LRLGE, GLRLM_LRHGE
Long-Run Low Gray-level Emphasis or Long-Run High Gray-level Emphasis is the distribution of the long homogeneous runs with low or high grey-levels.

$GLRLM\_LRLGE$ = *Average over* 13 (*or* 4) *directions*$\left(\frac{1}{H}\sum_i\sum_j \frac{GLRLM(i,j)\cdot j^2}{i^2}\right)$
$GLRLM\_LRHGE$ = *Average over* 13 (*or* 4) *directions*$\left(\frac{1}{H}\sum_i\sum_j GLRLM(i,j)\cdot i^2\cdot j^2\right)$

2.5 GLRLM_GLNUr, GLRLM_RLNU
Gray-Level Non-Uniformity for run or Run Length Non-Uniformity is the nonuniformity of the grey-levels or the length of the homogeneous runs.
$GLRLM\_GLNUr$= *Average over* 13 (*or* 4) *directions*$\left(\frac{1}{H}\sum_i\left(\sum_j GLRLM(i,j)\right)^2\right)$
$GLRLM\_RLNU$= *Average over* 13 (*or* 4) *directions*$\left(\frac{1}{H}\sum_j(\sum_i GLRLM(i,j))^2\right)$

2.6 GLRLM_RP
Run Percentage measures the homogeneity of the homogeneous runs.
$GLRLM\_RP$ = *Average over* 13 (*or* 4) *directions*$\frac{H}{\sum_i\sum_j(j\cdot GLRLM(i,j))}$

3. NGLDM
The neighborhood grey-level different matrix (NGLDM) [Amadasum1989] corresponds to the difference of grey-levels between one voxel and its 26 neighbours in 3 dimensions (8 in 2D). Three texture indices can be computed from this matrix. An element (i, 1) of NGLDM corresponds to the probability of occurrence of level i and an element (i, 2) is equal to:
$$NGLDM(i,2)=\sum_p\sum_q\begin{cases}|\bar{M}(p,q)-i| \text{if } I(p,q)=i\\ 0 \text{ else}\end{cases}$$
where $\bar{M}(p,q)$ is the average of intensities over the 26 neighbour voxels of voxel(p, q).

3.1 NGLDM_Coarseness
is the level of spatial rate of change in intensity
$NGLDM\_Coarseness$=$\frac{1}{\sum_i NGLDM(i,1)\cdot NGLDM(i,2)}$

3.2 NGLDM_Contrast
is the intensity difference between neighbouring regions.
$NGLDM\_Contrast$=$\left[\sum_i\sum_j NGLDM(i,1)\cdot NGLDM(j,1)\cdot(i-j)^2\right]\cdot\frac{\sum_i NGLDM(i,2)}{E\cdot G\cdot(G-1)}$
where E corresponds to the number of voxels in the Volume of Interest and G the number of grey-levels.

3.3 NGLDM_Busyness
is the spatial frequency of changes in intensity.
$NGLDM\_Busyness$=$\frac{\sum_i NGLDM(i,1)\cdot NGLDM(i,2)}{\sum_i\sum_j |(i\cdot NGLDM(i,1)-j\cdot NGLDM(j,1))|}$
with $NGLDM(i,1)\neq 0$, $NGLDM(j,1)\neq 0$

4. GLCM

The grey level co-occurrence matrix (GLCM) [Haralick] takes into account the arrangements of pairs of voxels to calculate textural indices. The GLCM is calculated from 13 different directions in 3D with a d-voxel distance ($\|\vec{d}\|$) relationship between neighboured voxels. The index value is the average of the index over the 13 directions in space (X, Y, Z). Seven textural indices are computed from this matrix. An entry (i, j) of GLCM for one direction is equal to:
$$GLCM_{\Delta x,\Delta y}(i,j)=\frac{1}{Pairs_{ROI}}\sum_{p=1}^{N-\Delta x}\sum_{q=1}^{M-\Delta y}\begin{cases}1 \text{ if } (I(p,q)=i,\ \ I(p+\Delta x,q+\Delta y)=j)\\ \text{ and } I(p,q), I\,(p+\Delta x,\ \ q+\Delta y)\in ROI\\ 0 \text{ otherwise}\end{cases}$$

where I(p, q) corresponds to voxel (p, q) in an image (I) of size N × M. The vector $\vec{d}$= (Δx,Δy) covers the 4 directions

(D1, D2, D3, D4) in 2D space or 13directions (D1, D2, ..., D13) in 3D space and $Pairs_{ROI}$ corresponds to the number of all voxel pairs belonging to the region of interest (ROI).The GLCM describes the distribution of co-occurring pixel values at a given offset.

4.1 GLCM_Homogeneity

is the homogeneity of grey-level voxel pairs.

GLCM_Homogeneity= *Average over* 13 (*or* 4) *directions*$\left(\sum_i \sum_j \frac{GLCM(i,j)}{1+|i-j|}\right)$

4.2 GLCM_Energy

also called Uniformity or Second Angular Moment, is the uniformity of grey-level voxel pairs.

GLCM_Energy= *Average over* 13 (*or* 4) *directions*$\left(\sum_i \sum_j GLCM(i,j)^2\right)$

4.3 GLCM_Correlation

is the linear dependency of grey-levels in GLCM.

GLCM_Correlation= *Average over* 13 (*or* 4) *directions*$\left(\sum_i \sum_j \frac{(i-\mu_i)\cdot(j-\mu_j)\cdot GLCM(i,j)}{\sigma_i \cdot \sigma_j}\right)$

where$\mu_i$or $\mu_j$corresponds to the average on row *i* or column *j* and $\sigma_i$and $\sigma_j$correspond to the variance on row *i* or column *j*

4.4 GLCM_Entropy_log10

is the randomness of grey-level voxel pairs.

*GLCM_Entropy_log*10 = *Average over* 13 (*or* 4) *directions*$\left(-\sum_i \sum_j GLCM(i,j) \cdot \log 10(GLCM(i,j)+\epsilon)\right)$. Where $\varepsilon=2e^{-16}$

GLCM_Entropy_log2 is the randomness of grey-level voxel pairs.

GLCM_Entropy_log2= *Average over* 13 (*or* 4) *directions*$\left(-\sum_i \sum_j GLCM(i,j) \cdot \log 2(GLCM(i,j)+\epsilon)\right)$. Where $\varepsilon=2e^{-16}$

4.5 GLCM_Contrast

also called Variance or Inertia, is the local variations in the GLCM

GLCM_Contrast= *Average over* 13 (*or* 4) *directions*$\left(\sum_i \sum_j (i-j)^2 \cdot GLCM(i,j)\right)$

4.6 GLCM_Dissimilarity

is the variation of grey-level voxel pairs.

GLCM_Dissimilarity= *Average over* 13 (*or* 4) *directions*$\left(\sum_i \sum_j |i,j| \cdot GLCM(i,j)\right)$
